# In search for biomarkers and potential drug targets for uterine serous endometrial cancer

**DOI:** 10.1007/s00432-021-03566-x

**Published:** 2021-03-23

**Authors:** Giorgia Dinoi, Andrea Mariani, Enrica Martinelli, Alessandra Ciucci, Gian Franco Zannoni, Amy L. Weaver, Gary L. Keeney, George Vasmatzis, Panos Z. Anastasiadis, Francesco Fanfani, Giovanni Scambia, Daniela Gallo

**Affiliations:** 1 Gynecology Oncology Unit, Department of Woman, Child and Public Health, Fondazione Policlinico Universitario A. Gemelli IRCCS, Largo A. Gemelli, 8, 00168, Rome, Italy; 2 Division of Gynaecologic Surgery, Department of Obstetrics and Gynaecology, Mayo Clinic, Rochester, MN USA; 3 Department of Life Sciences and Public Health, Section of Gynaecology and Obstetrics, Università Cattolica del Sacro Cuore, Rome, Italy; 4 Unit of Translational Medicine for Woman and Child Health, Department of Woman, Child and Public Health, Fondazione Policlinico Universitario A. Gemelli IRCCS, Rome, Italy; 5 Gynecopathology and Breast Pathology Unit, Department of Woman, Child and Public Health, Fondazione Policlinico Universitario A. Gemelli IRCCS, Rome, Italy; 6 Division of Clinical Trials and Biostatistics, Department of Quantitative Health Sciences, Mayo Clinic, Rochester, MN USA; 7 Division of Anatomic Pathology, Mayo Clinic Rochester, Rochester, MN USA; 8 Department of Molecular Medicine, Department of Laboratory Medicine & Pathology, Mayo Clinic, Rochester, MN USA; 9 Department of Cancer Biology, Mayo Clinic, Jacksonville, FL USA

**Keywords:** Serous endometrial cancer, Uterine malignancy, Biomarkers, Immunochemistry, Molecular profiling, Drug targets

## Abstract

**Objective:**

Serous endometrial cancer (USC) is a challenging malignancy associated with metastasis, recurrence and poor outcome. To identify clinically relevant prognostic biomarkers, we focused on a panel of proteins selected after a comprehensive literature review, for tumour profiling of a homogeneous cohort of USC patients.

**Methods:**

Protein levels and localization were assessed by immunohistochemistry analysis in 36 hysterectomy samples. Tissue sections were stained with the following antibodies: Aurora A, phospho (T288) Aurora A, BRCA1, CHK1, CIP2A, Cyclin B1, Cyclin E, E2F-1, phospho (S364) E2F-1, FBXW7, FOXM1, phospho (S9) GSK3Beta, PLK1, phospho (T210) PLK1, PPP2R1B, p73, RAD51. Each marker was evaluated as a continuously-scaled variable for association with disease progression and death, using Cox proportional hazards models. The sample consisted of 36 patients with USC, half with stage III or IV disease.

**Results:**

Results showed that higher CHK1 (Checkpoint kinase 1) expression was associated with a decreased risk of progression and death, after adjusting for stage. Interestingly, analysis of a TCGA data set of 109 USC patients corroborates our results showing a favourable prognostic role of CHEK1 after adjusting for stage. Higher FBXW7 (F-box and WD repeat domain containing 7) expression and higher cytoplasmic expression of PPP2R1B (Protein Phosphatase 2 A, Scaffold Subunit Abeta) were each associated with a decreased risk of progression, after adjusting for stage.

**Conclusions:**

In conclusion, results from the present study identify new clinically relevant biomarkers and potential drug targets for uterine serous endometrial cancer.

**Supplementary Information:**

The online version contains supplementary material available at 10.1007/s00432-021-03566-x.

## Introduction

Endometrial cancer (EC) is the most common gynaecological cancer in the developed world and the fourth most common cancer in women (Gentry-Maharaj and Karpinskyj 2020). Based on differences in histology and clinical outcomes, endometrial cancers have long been divided into the estrogen-dependent type I (endometrioid adenocarcinoma) with a favourable prognosis, and the estrogen-independent type II (predominantly serous and clear cell carcinoma) (Bokhman 1983; Dedes et al. 2011). Approximately 80–90% of EC are endometrioid-type carcinomas, and 2–10% are serous endometrial carcinomas (USC) (Dedes et al. 2011). Although these latter account for only a small percentage of EC, as high as 40% of EC deaths are attributed to USC (Hamilton et al. 2008; Del Carmen et al. 2012). This highly aggressive behaviour is mainly related to its tendency to metastasize, even when the primary tumour is small (Hamilton et al. 2006). Currently, the standard of care for most patients with USC includes surgery and platinum-based adjuvant chemotherapy with or without radiotherapy. Indeed, given its distinct biological behaviour, USC is typically excluded from large clinical trials (Keys et al. 2004; Nout et al. 2010).

During the last decade, we have significantly increased our understanding of biological pathways implicated in disease development and progression. Specifically, USC were found to harbour a specific genomic signature, which is different from other EC histologic subtypes, and it is partially overlapping with high-grade serous ovarian carcinoma (HGSOC) and basal-like breast cancer (Kuhn et al. 2012; Le Gallo et al. 2012; Cancer Genome Atlas Research Network 2013). Mutations in the *TP53* (Tumor protein p53) tumour suppressor gene and/or stabilization of the p53 protein were the most frequent molecular aberrations in serous carcinomas, occurring at frequencies in excess of 85%. Notably, however, the mutation frequency of five genes [PIK3CA (Phosphatidylinositol-4,5-Bisphosphate 3-Kinase Catalytic Subunit Alpha), PIK3R1 (phosphoinositide-3-kinase regulatory subunit 1), PTEN (phosphatase and tensin homolog), PPP2R1A (protein phosphatase 2 scaffold subunit Aalpha) and FBXW7 (F-box and WD repeat domain containing 7)] was dramatically higher in USC as compared to serous ovarian cancer (Cancer Genome Atlas Research Network 2013). These five genes are part of the PI3K (phosphatidylinositol 3-kinase)-AKT (protein kinase B)-FBXW7 pathway. The FBXW7 tumour suppressor is a component of the FBXW7-SKP1 (S-phase kinase-associated protein 1)-CUL1 (Cullin-1) ubiquitin ligase complex, which mediates the ubiquitination of the protein products of multiple oncogenes, including cyclin E, AURKA (Aurora A kinase), PLK1 (Polo-like kinase-1) and FOXM1 (Forkhead box protein M1) among the others (Galindo-Moreno et al. 2020). Glycogen synthase kinase-3 (GSK3)-mediated phosphorylation of the targeted proteins is a necessary step for subsequent FBXW7-mediated ubiquitylation and proteasomal degradation (Hoxhaj and Manning 2020). GSK3 is a ubiquitously expressed protein kinase that exists in two isoforms, α and β. The protein is active under basal conditions and is inhibited in response to growth factors and insulin, via AKT-mediated phosphorylation. GSK3 and FBXW7 thus act in concert to regulate ubiquitination of many important factors associated with cell division and growth.

Pathway analysis and clustering of the endometrial tumours in the TCGA data set also revealed that dysregulation of mitotic processes is a frequent occurrence in USC (Cancer Genome Atlas Research Network 2013). In this context, it is worth noting that numerous studies have established a crucial role for E2F-1 (E2F transcription factor 1) in the control of cellular proliferation, through transcriptional activation of genes important for cell-cycle progression, such as cyclin B1 and cyclin E (Hallstrom et al. 2008). Therefore, E2F-1 may have profound implications in USC, acting as a master regulator of cell fate. In line with this concept, E2F-1 has been identified as a prognostic biomarker in endometrial cancer, being mostly expressed in the serous histotype (Alkushi et al. 2007). Actually, E2F-1 has been shown to induce both proliferation and apoptosis, being the balance of these events regulated by the PI3K/AKT signalling, that would selectively block the expression of genes in the apoptotic program, but not in the proliferative program (Hallstrom et al. 2008). *TP73* (Tumor protein p73) is a target of E2F-1 apoptotic program and altered *TP73* expression (due to aberrant DNA methylation) is considered a predictor of USC histology (Seeber et al. 2010). Notably, after DNA damage, p73 induction is regulated by the checkpoint kinases CHK1 and CHK2, through E2F-1 stabilization, in a pathway central to p53-independent apoptosis (Urist et al. 2004). Therefore, this signalling may be a major determinant of anti-cancer drug efficacy in USC patients carrying *TP53* mutations. Overall, these data suggest that molecular pathways converging on p73 may play important roles in driving disease outcome.

TCGA data also demonstrated that up to 40% of type II EC tumours are associated with heterozygous missense mutations in PPP2R1A, an established tumour suppressor gene encoding the Aα subunit of PP2A (Protein Phosphatase 2A), one of the major cellular serine–threonine phosphatases, involved in the regulation of PI3K/AKT pathway (Kuo et al. 2008; Remmerie and Janssens 2019). It has been recently shown that also PPP2R1B (the gene encoding for the β isoform of subunit A) is mutated in human neoplasm, with a functional inactivation of the protein that may promote tumorigenesis, through its role in cell-cycle regulation and cellular growth control (Calin et al. 2000). The inclusion of both isoforms of subunit A in the genes mutated in human cancer support the potential role of PP2A in human tumorigenesis via a mechanism of inactivation of the phosphatase activity (Calin et al. 2000). In line with these findings, overexpression of CIP2A (Cellular Inhibitor of PP2A, also called p90), an endogenous PP2A-inhibitory protein, has been associated to worse outcome in gynaecologic cancers (Remmerie and Janssens 2019).

Finally, recent data supported the view that homologous recombination deficiency (HRD) occurs in endometrial cancers and is largely restricted to non-endometrioid, *TP53*-mutant endometrial cancers, the spectrum of germline mutations detected including BRCA1/2 (breast cancer type 1/2 susceptibility protein) and RAD51 (DNA repair protein RAD51 homolog 1) among others (Ring et al. 2016; De Jonge et al. 2019).

Based on the above-mentioned, well-known or emerging evidence of molecular determinants of cancer development, we selected a panel of proteins for tumour profiling of a homogeneous cohort of USC patients, to identify clinically relevant prognostic biomarkers. Results obtained provide new perspective and potential strategies for drug target discovery in USC therapy.

## Materials and methods

### Patients

We analysed a retrospective series of patients with pathological confirmed diagnosis of serous endometrial cancer, who underwent surgery between January, 2009 and December, 2012 at Mayo Clinic (Rochester, Minnesota). Approval was provided by the Institutional Review Board (IRB) at Mayo Clinic. Clinical information was obtained from the existing medical records in accordance with the institutional guidelines. Data abstracted from the medical records included demographic (e.g., age at surgery, BMI) as well as surgical and pathological details [e.g., surgical approach, histology, International Federation of Gynecology and Obstetrics (FIGO) grade and stage, tumour diameter, lymph-vascular space invasion (LVSI), myometrial invasion, peritoneal cytology, pelvic and para-aortic lymph nodes status]. Patients who did not give research authorization, with synchronous cancers, or who underwent neoadjuvant chemotherapy were excluded. Formalin-fixed paraffin-embedded (FFPE) tissue blocks were cut as consecutive sections and slides sent to the Gynaecology Oncology Unit, Fondazione Policlinico A. Gemelli IRCCS, Rome, Italy, for immunohistochemical staining and analysis of the selected protein panel.

### Immunohistochemistry

A board-certified gynaecologic pathologist (GFZ) reviewed all tumours to confirm the diagnosis of USC. Tissue sections were stained with the following antibodies: Aurora A, phospho (T288) Aurora A, BRCA1, CHK1, CIP2A/p90, Cyclin B1, Cyclin E, E2F-1, phospho (S364) E2F-1, FBXW7, FOXM1, phospho (S9) GSK3Beta, PLK1, phospho (T210) PLK1, PPP2R1B, p73 and RAD51.

The process of deparaffinization, rehydration and epitope retrieval of tissue specimens was performed with low or high pH Target Retrieval Solution (Agilent Technologies, Santa Clara, CA, USA) in DAKO PT Link module (Agilent Technologies). The endogenous peroxidase was blocked with 3% H_2_O_2_ for 5 min. To reduce non-specific binding, sections were incubated with 20% normal goat serum for 30 min, at room temperature, and then with primary antibody in a humidified chamber. Conditions for antigen retrieval, incubation times, and the primary antibodies used are described in Table S1 (see Supplement). Sections were incubated with the secondary antibody, anti-mouse/rabbit EnVision System-HRP (Dako, Agilent) for 30 min, at room temperature. The slides were developed with diaminobenzidine (DAB substrate System, Dako, Agilent), counterstained with Mayer’s Haematoxylin, dehydrated in ethanol and xylene and mounted. Staining without primary antibody was used to validate the specificity of the secondary antiserum, while a section from a tissue known to express the protein of interest was used as positive control. Expression was evaluated by considering the percentage of cells exhibiting immunoreaction, as well as the localization of signalling (nuclear and/or cytoplasmic). Graphical analysis was performed using GraphPad Prism 6 (GraphPad Software, Inc. La Jolla, CA, USA). Two independent observers (GFZ and EM), who were unaware of the patients’ outcome, evaluated immunostained tissue sections.

### Statistical analysis of the study sample

Baseline characteristics were summarized using standard descriptive statistics: frequencies and percentages for categorical variables while mean and standard deviation (SD) or standard error of mean (SEM) for continuous variables. Duration of follow-up was calculated from the date of the surgery to the date of progression (or date of last relevant clinical follow-up for those without progression), and from the date of the surgery to the date of death (or date of last known vital status for those not deceased). Progression-free survival (PFS) and overall survival (OS) were estimated using the Kaplan–Meier method. Univariate Cox proportional hazards models were fit to evaluate the association of each continuously-scaled marker with progression or death, respectively. Additional models were fit adjusting for stage (FIGO stage III/IV vs. I/II). Associations were summarized using the hazard ratio per 10 unit increase in each marker and corresponding 95% confidence interval (CI) estimated from the models. Data were analysed using the SAS version 9.4 software package. *p* Values were two-sided, with *p* < 0.05 considered as significant.

### Bioinformatics analysis of TCGA database

Biomarkers with statistically significant associations with both progression and death, after adjusting for stage, were further investigated through an external dataset. Uterine Corpus Endometrial Carcinoma (UCEC) data were retrieved from the publicly available and curated database cBioPortal (cBioPortal for cancer genomics, www.cbioportal.org, 2020), extracting clinical and gene expression features from the TCGA PanCancer Atlas dataset (Cerami et al. 2012; Gao et al. 2013). In particular, among the 529 cases, only 109 Uterine Serous Carcinoma/Uterine Papillary Serous Carcinoma cases were available. Gene expression levels were obtained from the mRNA Expression RSEM (Batch normalized from Illumina HiSeq_RNASeqV2) dataset and matched to the clinical features. The best cutoff for gene expression values was chosen based on the results of Cutoff Finder analyses implemented in R v3.6.1 software environment (R Core Team, 2019). The best cutoff value was used to categorize patients into low- and high-expression value groups. The prognostic effect of the clinical and molecular parameters on the risk of death was evaluated using the Cox proportional hazard model. Statistical analysis was performed using Stata software (StataCorp. 2011. Stata Statistical Software: Release 12. College Station, TX: StataCorp LP). In addition, we used cBioPortal online platform to predict co-expressed genes with the investigated biomarkers among the 109 USC samples of the TCGA data set.

## Results

During January 2009 through December 2012, a total of 93 consecutive patients diagnosed with serous or mixed serous EC underwent primary surgery at our institution and met the following inclusion criteria: research authorization, no synchronous cancer or previous neoadjuvant therapies. Of these patients, 34 had mixed serous histology and 59 had serous histology. Among these 59 women, the results herein are based on the 36 patients whose specimens were available and there was adequate tissue in the paraffin block to analyze multiple markers with immunohistochemistry.

### Clinicopathological characteristics

Clinicopathological characteristics of the 36 patients are summarized in Table 1. Mean age of patients at the time of surgery was 72.2 years (SD 9.0; 54.9–89.8 years). Half (50%) of the patients had advanced stage of disease (from FIGO stage IIIA to stage IVB). Tumour diameter, available for 34 patients, was > 2 cm in 91.2% of cases; lymph-vascular space invasion (LVSI) was present in six patients (16.7%). More than two-third of patients presented myometrial invasion (29 patients, 80.6%). Of the 30 patients who had a pelvic lymphadenectomy performed, 10 (33.3%) had positive pelvic nodes. Of the 30 patients who had a para-aortic lymphadenectomy, 6 (20.0%) had positive para-aortic nodes. Twenty-four (66.4%) patients underwent any adjuvant treatment.

**Table 1:** Clinicopathological characteristics of uterine serous carcinomas (USC) patients

Characteristics^†^	Result
All cases	36
Age at surgery (years)	
Mean (SD)	72.2 (9.0)
Range	54.8–89.8
BMI (kg/m^2^)	
Mean (SD)	32.8 (8.9)
Range	15.8–60.5
Surgical approach	
Laparotomy	28 (77.8%)
Robotic	6 (16.7%)
Vaginal/robotic	1 (2.8%)
Vaginal	1 (2.8%)
FIGO stage	
IA	15 (41.7%)
IB	2 (5.6%)
II	1 (2.8%)
IIIA/B	1 (2.8%)
IIIC1	5 (13.9%)
IIIC2	5 (13.9%)
IV	7 (19.4%)
Tumour diameter	
≤ 2 cm	3/34 (8.8%)
> 2 cm	31/34 (91.2%)
LVSI	
No	30 (83.3%)
Yes	6 (16.7%)
Myometrial invasion	
No	7 (19.4%)
Yes	29 (80.6%)
Peritoneal cytology	
Positive	7/33 (21.2%)
Negative	26/33 (78.8%)
Lymphadenectomy	
No	5 (13.9%)
Pelvic and para-aortic	29 (80.6%)
Pelvic only	1 (2.8%)
Paraaortic only	1 (2.8%)
Positive lymph nodes	
Pelvic positive	10/30 (33.3%)
Paraaortic positive	6/30 (20.0%)
Adjuvant therapy	
None	7 (19.4%)
VBT only	8 (22.2%)
Chemo ± VBT	7 (19.4%)
Chemo ± EBRT ± VBT	9 (25.0%)
Unknown	5 (13.9%)

*BMI* body mass index, *LVSI* lymphovascular space invasion, *VBT* vaginal brachytherapy, *EBRT* external beam radiation therapy

^†^Results reported as *N* (%) unless otherwise noted

### Clinical outcomes

During the follow-up period, progression and death of disease were observed in 18 and 23 patients, respectively. Based on the Kaplan–Meier method the median PFS and OS was 3.3 and 5.0 years, respectively. The median duration of follow-up was 5.3 years (IQR 0.4–7.4 years) among those without a progression and 7.4 years (IQR, 6.9–8.1 years) among the non-deceased patients.

**Fig. 1:**
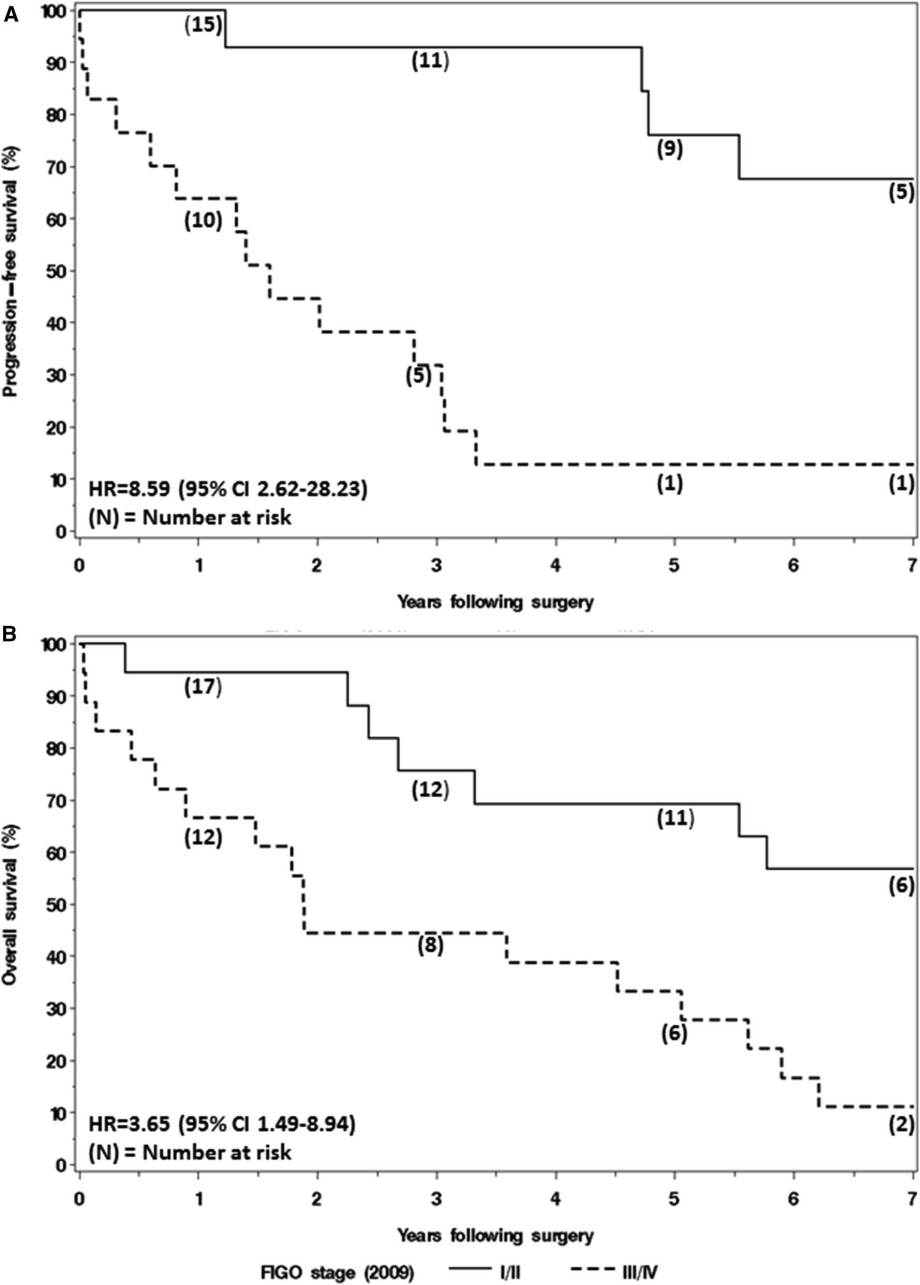
Kaplan–Meier curves for progression-free survival (**a**) and overall survival (**b**), according to stage

The risk of progression was significantly higher among patients with advanced stage (III or IV) disease with a hazard ratio of 8.59 (95% CI 2.62–28.23; *p* < 0.001, Fig. 1a). In addition, the risk of death (due to any cause) was significantly higher among patients with advanced stage (III or IV) disease with a hazard ratio of 3.65 (95% CI 1.49–8.94; *p* = 0.005, Fig. 1b).

### Expression of molecular markers in the study cohort

**Fig. 2:**
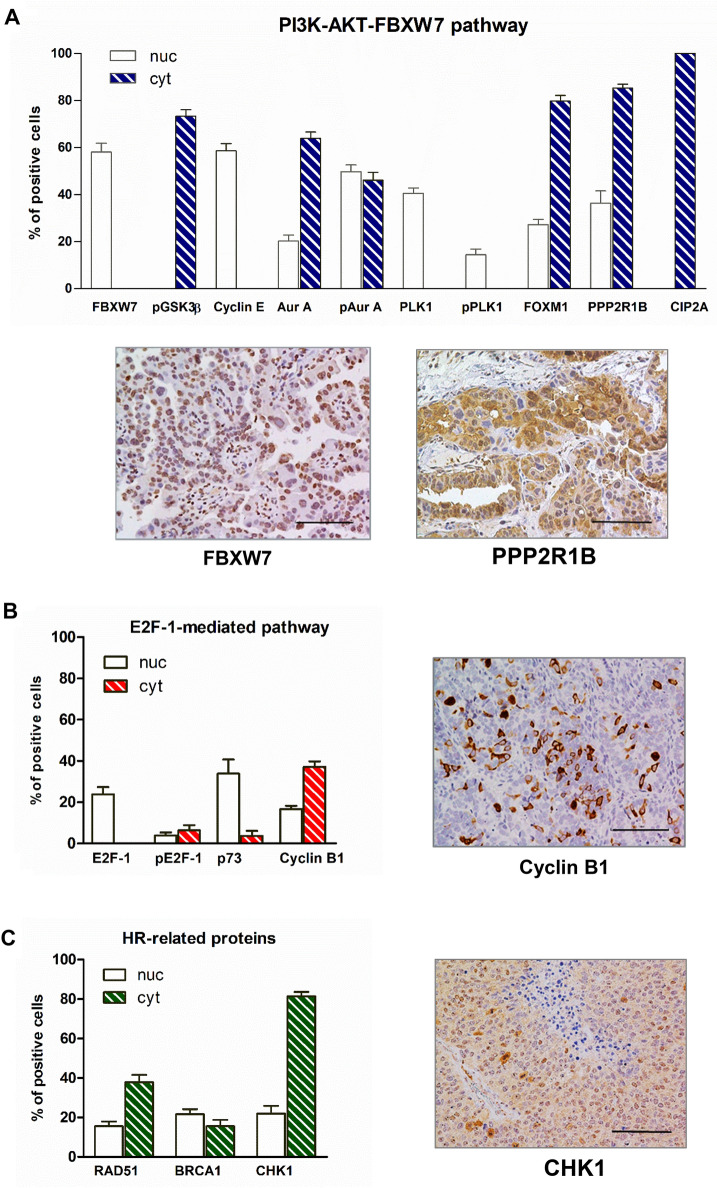
Immunohistochemistry-based protein profiling of uterine serous carcinomas (UCS) patients. Bar charts show protein expression grouped in PI3K-AKT-FBXW7 pathway (**a**), E2F-1-mediated pathway (**b**) and HR-related proteins (**c**). The bars represent the mean ± SEM of positive cells for the indicated proteins in the nuclear (nuc) and/or cytoplasmic (cyt) compartment (*n* = 36). *P* indicates the phosphorylated form of the protein. Adjacent to each graph are shown representative immunohistochemical pictures of proteins significantly affecting PFS or OS when adjusting for stage (magnification 20X)

Figure 2 illustrates the mean percentage of expression (with SEM) for each marker in the nuclear and/or cytoplasmic cellular compartments. Accurate immunohistochemical analysis of tissue sections showed that FBXW7, Cyclin E and PLK1 were expressed in all samples and almost exclusively localized in the nuclear compartment. PLK1 is an essential mitotic kinase regulating multiple aspects of the cell division process. Activation of PLK1, driving cells into mitosis, requires phosphorylation of a conserved threonine residue (Thr 210), a process mainly mediated by Aurora A (Bruinsma et al. 2017); p(T210)PLK1 was expressed in the nuclear compartment of 23 out of 36 samples, this suggesting differences in pathway activation among patients. On the other hand, staining of Aurora A and active Aurora A phosphorylated on Thr288 was found in all samples in the nuclear and/or cytoplasmic compartments. Immunohistochemistry also showed that the expression of pGSK3β, FOXM1, PPP2R1B and CIP2A was predominantly evident in the cytoplasm of all analysed sections. It is worth noting that FOXM1, a critical proliferation-associated transcription factor closely involved with the processes of cell proliferation, self-renewal and tumorigenesis (Liao et al. 2018) was present as nuclear staining in all patients, although at a different extent among samples.

Expression of E2F-1 was exclusively nuclear, with low activation, as demonstrated by the negative/low levels of p(S364) E2F-1 detected in the majority of the sections. Most samples were negative for p73 (21 out of 36); however, when p73 was expressed, the protein was localized in the nucleus. Cyclin B1 was mainly localized into the cytoplasmic compartment, although a low nuclear staining was also evident in most samples.

All sections analysed showed intense CHK1 staining in the cytoplasmic localization, with the majority also exhibiting low-medium nuclear expression. Finally, low/medium BRCA1 and RAD51 levels were detected in most sections.

### Associations between biomarker levels and clinical outcomes

The association of each biomarker with the risk of recurrence and death, respectively, was evaluated univariately and after adjusting for stage (i.e. FIGO stage III/IV vs. I/II). The hazard ratios (per 10-unit increase) are presented in Tables 2and 3. Consistently high-expression levels of CIP2A were detected in all samples and, therefore, this protein was not evaluated in this analysis. Likewise, due to the low number of positive samples (8 out of 36), p(S364) E2F-1 was also not included in outcome analysis.

**Table 2:** Univariate analysis of markers evaluated for an association with progression in uterine serous carcinomas (USC) patients

Marker	Univariate analysis, unadjusted		Univariate analysis, adjusted for stage^†^	
	HR (95% CI)	*P*^‡^	HR (95% CI)	*P*^‡^
FBXW7 nuc	0.85 (0.71,1.01)	0.07	0.80 (0.66, 0.97)	0.024
pGSK3β cyt	1.01 (0.74,1.37)	0.96	1.09 (0.75, 1.57)	0.67
Cyclin E nuc	0.96 (0.77,1.19)	0.69	1.05 (0.85, 1.29)	0.64
Aurora A nuc	1.14 (0.82,1.59)	0.44	0.89 (0.65, 1.22)	0.46
Aurora A cyt	1.29 (0.97,1.72)	0.08	1.22 (0.91, 1.64)	0.18
pAurora A nuc	0.93 (0.73,1.20)	0.59	0.92 (0.66, 1.29)	0.63
pAurora A cyt	1.07 (0.83,1.38)	0.60	0.99 (0.78, 1.25)	0.91
PLK1 nuc	0.77 (0.50,1.18)	0.23	0.97 (0.62, 1.54)	0.91
pPLK1 nuc	0.86 (0.60,1.23)	0.41	0.82 (0.55, 1.20)	0.30
FOXM1 nuc	1.03 (0.71,1.48)	0.90	1.02 (0.71, 1.46)	0.92
FOXM1 cyt	1.39 (0.99,1.94)	0.06	1.26 (0.89, 1.77)	0.19
PPP2R1B nuc	0.93 (0.80,1.10)	0.40	0.92 (0.77, 1.10)	0.37
PPP2R1B cyt	0.85 (0.53,1.36)	0.49	0.59 (0.36, 0.96)	0.035
E2F-1 nuc	0.89 (0.71,1.12)	0.32	0.78 (0.58, 1.05)	0.11
p73 nuc	1.02 (0.90,1.14)	0.80	1.00 (0.89, 1.12)	0.97
Cyclin B1 nuc	1.04 (0.63,1.70)	0.89	1.44 (0.82, 2.51)	0.20
Cyclin B1 cyt	0.80 (0.56,1.13)	0.21	0.88 (0.59, 1.32)	0.54
RAD51 cyt	0.91 (0.74,1.11)	0.34	0.87 (0.69, 1.10)	0.25
RAD51 nuc	0.97 (0.69,1.36)	0.87	0.91 (0.63, 1.31)	0.61
BRCA1 nuc	1.48 (1.05,2.09)	0.025	1.08 (0.73, 1.60)	0.72
BRCA1 cyt	1.12 (0.89,1.41)	0.35	0.93 (0.74, 1.17)	0.53
CHK1 nuc	0.67 (0.50,0.90)	0.008	0.57 (0.39, 0.82)	0.002
CHK1 cyt	0.91 (0.66,1.26)	0.57	0.56 (0.36, 0.87)	0.010

*PFS* progression-free survival, *HR* hazard ratio per 10 unit increase in each marker, *CI* confidence interval, *Nuc* nuclear compartment, *Cyt* cytoplasmic compartment

^†^Stage was categorized as III/IV vs. I/II when included as a covariate in each regression model

^‡^*P* values were derived from the Cox proportional hazards regression model

**Table 3:** Univariate analysis of markers evaluated for an associations with death (due to any cause) in uterine serous carcinomas (USC) patients

Marker	Univariate analysis, unadjusted		Univariate analysis, adjusted for stage^†^	
	HR (95% CI)	*P*^‡^	HR (95% CI)	*P*^‡^
FBXW7 nuc	0.93 (0.79,1.10)	0.38	0.94 (0.79, 1.11)	0.44
pGSK3β cyt	0.85 (0.65,1.10)	0.22	0.80 (0.60, 1.06)	0.12
Cyclin E nuc	0.95 (0.78,1.16)	0.63	1.00 (0.83, 1.21)	0.98
Aurora A nuc	1.43 (1.09,1.89)	0.011	1.25 (0.96, 1.64)	0.10
Aurora A cyt	1.13 (0.88,1.44)	0.34	1.00 (0.78, 1.28)	0.99
pAurora A nuc	0.93 (0.74,1.17)	0.54	0.93 (0.71, 1.21)	0.57
pAurora A cyt	1.05 (0.85,1.31)	0.63	1.02 (0.85, 1.24)	0.81
PLK1 nuc	0.80 (0.56,1.16)	0.24	0.97 (0.66, 1.44)	0.89
pPLK1 nuc	0.98 (0.73,1.33)	0.92	1.03 (0.76, 1.40)	0.84
FOXM1 nuc	0.82 (0.58,1.14)	0.24	0.78 (0.56, 1.08)	0.13
FOXM1 cyt	1.08 (0.83,1.41)	0.57	0.96 (0.73, 1.26)	0.75
PPP2R1B nuc	1.00 (0.88,1.14)	0.97	1.02 (0.89, 1.17)	0.78
PPP2R1B cyt	1.06 (0.66,1.70)	0.80	0.82 (0.51, 1.30)	0.39
E2F-1 nuc	0.90 (0.74,1.10)	0.29	0.84 (0.66, 1.06)	0.13
p73 nuc	1.07 (0.96,1.18)	0.21	1.04 (0.94, 1.15)	0.47
Cyclin B1 nuc	1.18 (0.76,1.82)	0.46	1.55 (0.96, 2.50)	0.07
Cyclin B1 cyt	0.75 (0.54,1.03)	0.08	0.76 (0.53, 1.08)	0.13
RAD51 cyt	0.89 (0.74,1.07)	0.21	0.87 (0.71, 1.06)	0.17
RAD51 nuc	1.06 (0.79,1.41)	0.72	1.07 (0.79, 1.44)	0.67
BRCA1 nuc	1.31 (0.98,1.74)	0.06	1.09 (0.79, 1.50)	0.59
BRCA1 cyt	1.07 (0.89,1.30)	0.48	0.91 (0.74, 1.12)	0.38
CHK1 nuc	0.76 (0.61,0.94)	0.013	0.75 (0.59, 0.96)	0.023
CHK1 cyt	0.92 (0.68,1.23)	0.56	0.66 (0.46, 0.96)	0.028

*OS* overall survival, *HR* hazard ratio per 10 unit increase in each marker, *CI* confidence interval, *Nuc* nuclear compartment, *Cyt* cytoplasmic compartment

^†^Stage was categorized as III/IV vs. I/II when included as a covariate in each regression model

^‡^*p* Values were derived from the Cox proportional hazards regression model

Focusing on the analysis adjusted for stage, we found that a higher expression of CHK1 both in the nuclear and cytoplasmic compartments was significantly associated with a decreased risk of progression (HR = 0.57 and 0.56, respectively). Likewise, higher nuclear FBXW7 and cytoplasmic PPP2R1B levels were significantly associated with a decreased risk of progression (HR = 0.80 and 0.59, respectively). CHK1 was the only biomarker significantly associated with death, and patients with high protein expression showed a reduced risk of death (HR = 0.75 and 0.66, for nuclear and cytoplasmic localization, respectively). Finally, cyclin B1, when overexpressed in the nucleus, was shown to be a negative prognostic factor for death (HR = 1.55, *p* = 0.07).

Interestingly higher nuclear BRCA1 expression was associated with an increased risk of progression (HR = 1.47, *p* = 0.025) and death (HR = 1.31, *p* = 0.06) upon univariate analysis. However, after adjusting for stage, both of these associations were greatly attenuated (HR = 1.08, *p* = 0.92; HR = 1.09, *p* = 0.59).

### Association of CHK1 expression to survival prognosis in TCGA cohort

To find confirmation for CHK1 as a favourable marker of outcome in USC patients, we interrogated the TCGA dataset by evaluating the association between CHEK1 (CHK1) mRNA levels and the risk of death in data from 109 patients, this latter being the first recommended clinical outcome endpoint for USC (Liu et al. 2018). We found that, after adjusting for tumour stage, CHK1 expression > 379.2 (“high CHK1”) was associated with a decrease in the risk of death (HR 0.35, 95% CI 0.16–0.77, *p* = 0.01). To gain insights into the signalling triggered by CHK1, we used cBioPortal (http://www.cbioportal.org/) to predict CHK1 co-expression genes among the 109 USC samples of the TCGA data set. Interestingly, we found that the protein-coding gene identified with the highest Spearman correlation value was EI24 (etoposide-induced gene 2.4) (Spearman’s correlation = 0.72, *p* < 0.001). This gene has been shown to play an important role in negative cell growth control, apoptosis and activation of autophagy (Zhao et al. 2012).

## Discussion

Results from the present study offer some important insights into USC, which is a rare aggressive subtype of endometrial cancer (Brooks et al. 2019).

The most interesting finding emerging from our investigation is the favourable prognostic role of CHK1 for both progression-free and overall survival, a result consistently observed for both the nuclear and the cytoplasmic protein fraction. Interestingly, analysis of a TCGA data set of 109 USC patients corroborates our results showing a favourable prognostic role of CHK1 after adjusting for stage. CHK1 is a key signal transducer in the DNA-damage response pathways, being implicated in the induction of cell-cycle arrest, DNA repair and apoptosis (Bartek and Lukas 2003). The protein is mainly expressed in the nucleus, but, following activation, it shuttles between the nucleus and the cytoplasm, regulating both nuclear and cytoplasmic checkpoints; importantly, cytoplasmic CHK1 does not support cell viability (Wang et al. 2012). Uncertainty exists in the literature about the role of CHK1 in cancer development and progression. Indeed, given the regulatory role it plays in DNA damage, CHK1 has long been recognized as a tumour suppressor. However, recent evidences indicate that CHK1 may contribute to tumour growth, thus representing a potential target in anti-cancer therapy. The notion that tumour cells (often p53-deficient and defective in G1 arrest), unlike normal cells, rely mainly on S or G2 checkpoints mediated by CHK1 to repair their damaged DNA and preserve their genomic integrity for basic viability, supports this view (Chen et al. 2006). In line with this concept, no homozygous loss-of-function mutation of CHK1 has been detected in a wide range of human tumours (Remmerie and Janssens 2019), an observation suggesting that cells with defective CHK1 are eliminated during tumorigenesis. Finally, the gene has been found overexpressed in variety of tumours and expression correlated with tumour grade and disease recurrence (Zhang and Hunter 2014). On the other hand, CHK1 frameshift mutations have been reported in genetically unstable colorectal and endometrial cancers, and these mutations might be involved in tumorigenesis, through a defect in response to DNA damage (Bertoni et al. 1999; Vassileva et al. 2002). Although these mutations predict for truncated CHK1 protein, their pathogenic implication is not evident since they have been always found as heterozygous aberrations. In keeping with our results, previous studies have also demonstrated that constitutive activation of CHK1, in the absence of DNA-damage, leads to cell-cycle arrest and eventually cell death (Wang et al. 2012; Zhang and Hunter 2014). Indeed, by querying the public TCGA USC sequencing data, we found that the top coding-gene associated with CHK1 was EI24, a putative tumour suppressor gene, whose reduced expression has been associated with the induction of EMT and tumour progression (Choi et al. 2013). Notably, EI24 has been also characterized as an E2F-1 target gene (Sung et al. 2013). Relevant to our results, Urist and colleagues (2004) demonstrated that CHK1 is required for induction of p73 following DNA damage and that E2F-1 is critical in this regulation. This implies that the CHK1–E2F-1–p73 pathway is central to p53-independent apoptosis, after DNA damage. Interestingly, literature data also unveiled Aurora A tumorigenic properties actually mediated by repression of CHK1 kinase activity, with a consequent impairment of the error-free homologous recombination pathway (Sourisseau et al. 2010). Overall, these findings might support the idea that in USC elevated constitutive levels of CHK1 play a protective role in disease development, through induction of permanent cell-cycle arrest and cell death. In addition, the outcome of CHK1 signalling following DNA-damaging therapies may also lead to increased apoptosis linked to p73 induction.

Besides CHK1, also FBXW7 and PPP2R1B were found to have a favourable prognostic role for progression, in analysis adjusted for stage. FBXW7 is a candidate driver gene somatically mutated in about 15–29% USC (Le Gallo et al. 2012; Cancer Genome Atlas Research Network 2013). Besides its role as tumour suppressor, emerging evidence suggest that lack of protein or loss-of-function mutations in FBXW7 confer resistance to antitubulin agents (Wertz et al. 2011), while sensitizing to HDAC inhibitors (Garnett et al. 2012). In this context, results of our study, if confirmed in a larger population, could have a clinical significance in guiding personalized therapy. With regard to PPP2R1B, our findings are in line with available data testifying a regulator function in a variety of cellular processes, including cell-cycle progression, whose alteration may be involved in tumorigenesis (Calin et al. 2000). However, to the best of our knowledge, no other evidences are available on its role as prognostic biomarkers in USC. Again, these findings could open new perspective and potential strategies for drug target discovery in USC therapy.

Results from the present study also suggest that Cyclin B1 is an unfavourable prognostic biomarker for death in USC. Cyclin B1 is a positive regulator of cell-cycle progression; protein overexpression reduces cell-cycle length and enables cells to override the G2 DNA damage checkpoint (Jin et al. 1998; Pomerening et al. 2005), finally leading to uncontrolled proliferation. At present, there are few studies regarding the role of Cyclin B1 in USC development and progression, although recent findings by Kwan and colleagues (Kwan et al. 2020) showed high Cyclin B1 expression levels in USC.

Finally, we also found that, at univariate analysis, higher nuclear BRCA1 expression was associated with an increased risk of progression and death. This result is in line with data from HGSOC, showing that patients with low BRCA1 expression had a more favourable outcome (Weberpals et al. 2011).

Beside the above-mentioned biomarkers, here we also give evidence that, although not having a role as prognostic factors, some proteins are expressed at high levels in USC tumours and specific pathways activated. In this respect, our findings therefore also provide hints for further studies specifically investigating those proteins found to be highly expressed in the disease and their potential value as new therapeutic targets.

Among the peculiar strengths of this study are that patients were managed in a tertiary centre, with well-documented electronic charts and dedicated personnel drawing up the historical EC database. In addition, the follow-up was sufficiently long to capture enough events, thus providing adequate statistical power to identify meaningful associations. Finally, all samples were treated and analysed with standard protocols. Besides the above-mentioned strengths, few limitations in our study need to be acknowledged. First, sample size was not large enough for detecting modest associations between biomarkers expression and the risk of progression or death. Moreover, pathologist-dependent interpretation of immunohistochemical staining might represent a setback, therefore, requiring all necessary steps to avoid any bias. Not least important, the retrospective nature of such a study design can lead to missing data.

In conclusion, results from the present study, although preliminary, highlight that molecular tumour profiling may provide a prognostic tool in serous endometrial cancer, representing an essential first step in drug discovery and development of personalized therapy.

## Supplementary Information

Below is the link to the electronic supplementary material.

Supplementary file1 (PDF 78 KB)
