# Supplementary material for: In search for biomarkers and potential drug targets for uterine serous endometrial cancer
Source: J Cancer Res Clin Oncol. 2021 Mar 23;147(6):1647–58. doi: 10.1007/s00432-021-03566-x (PMC8076151; doi:10.1007/s00432-021-03566-x)
Supplement: Supplementary file 1 — Supplementary file1 (PDF 78 KB) [file 432_2021_3566_MOESM1_ESM.pdf]

In Search for Biomarkers and Potential Drug Targets for Uterine Serous Endometrial Cancer.

Journal of Cancer Research and Clinical Oncology

Authors: Dinoi Giorgia[1,2], Mariani Andrea[2], Martinelli Enrica[3,4], Ciucci Alessandra[3,4], Zannoni Gian Franco[5], Weaver Amy[6], Gary L Keeney[7], Vasmatzis George[8], Anastasiadis Panagiotis Z[9], Fanfani Francesco[1], Scambia Giovanni[1§], Gallo Daniela[3,4]

Author Affiliations

[1]Gynecology Oncology Unit, Department of Woman, Child and Public Health, Fondazione Policlinico Universitario A. Gemelli IRCCS, Rome, Italy;

[2] Division of Gynaecologic Surgery, Department of Obstetrics and Gynaecology, Mayo Clinic, Rochester, Minnesota, USA;

[3]Department of Life Sciences and Public Health, Section of Gynaecology and Obstetrics, Università Cattolica del Sacro Cuore, Rome, Italy;

[4]Unit of Translational Medicine for Woman and Child Health, Department of Woman, Child and Public Health, Fondazione Policlinico Universitario A. Gemelli IRCCS, Rome, Italy;

[5]Gynecopathology and Breast Pathology Unit, Department of Woman, Child and Public Health, Fondazione Policlinico Universitario A. Gemelli IRCCS, Rome, Italy;

[6]Division of Biomedical Statistics and Informatics, Department of Health Sciences Research, Mayo Clinic, Rochester, Minnesota, USA;

[7]Division of Anatomic Pathology, Mayo Clinic Rochester, Rochester, Minnesota, USA;

[8]Department of Molecuar Medicine, Department of Laboratory Medicine & Pathology, Mayo Clinic, Rochester, Minnesota, USA;

[9]Department of Cancer Biology, Mayo Clinic Comprehensive Cancer Center, Jacksonville, Florida, USA.

§To whom correspondence should be addressed

Corresponding author

Giovanni Scambia

Gynecology Oncology Unit, Department of Woman, Child and Public Health, Fondazione Policlinico Universitario A. Gemelli IRCCS, Rome, Italy;

Largo A. Gemelli, 8, 00168, Rome, Italy

Tel. 0039-0630154979

Fax 0039-0630157063

E-mail: giovanni.scambia@policlinicogemelli.it

Table S1. List of Antibodies used in immunohistochemistry including specifies about antigen retrieval, working dilution and incubation.

Antigen	Source	Clone	Antigen retrieval	Working dilution	Incubation
Aurora A	Abcam	Polyclonal	High 20 min	1:200	4°C, ON
p(T288)Aurora A	Abcam	Polyclonal	High 20 min	1:200	4°C, ON
BRCA1	Calbiochem	MS110	Low 15 min	1:50	4°C, ON
CHK1	Abcam	Polyclonal	High 20 min	1:250	4°C, ON
CIP2A	Novusbio	Polyclonal	High 20 min	1:100	4°C, ON
Cyclin B1	Abcam	Y106	Low 20 min	1:200	4°C, ON
Cyclin E	SantaCruz	HE12	Low 20 min	1:50	4°C, ON
FBXW7	Abnova	3D1	Low 15 min	1:50	37°C, 1 h
E2F-1	SantaCruz	KH95	High 20 min	1:50	4°C, ON
p(S364)E2F-1	Abcam	Polyclonal	Low 15 min	1:800	4°C, ON
FOXM1	ThermoFisher	Polyclonal	High 20 min	1:750	4°C ON
p(S9)GSK3β	Abcam	EPR2286Y	Low 20 min	1:100	4°C, ON
PLK1	Cell Signal	208G4	High 20 min	1:50	4°C, ON
p(T210)PLK1	Abcam	EPNCIR167	Low 15 min	1:6000	RT, 0.5h
PPP2R1B	Novusbio	Polyclonal	Low 20 min	1:100	4°C, ON
P73	Abcam	EP436Y	High 20 min	1:200	4°C, ON
RAD51	ThermoFisher	3C10	High 30 min	1:100	37°C, 1 h

ON=overnight; RT=room temperature
